# Neurochemical phenotype and function of endomorphin 2-immunopositive neurons in the myenteric plexus of the rat colon

**DOI:** 10.3389/fnana.2014.00149

**Published:** 2014-12-16

**Authors:** Jun-Ping Li, Xi-Yu Wang, Chang-Jun Gao, Yong-Hui Liao, Juan Qu, Zhong-Yi He, Ting Zhang, Guo-Du Wang, Yun-Qing Li

**Affiliations:** ^1^Department of Anatomy, Histology and Embryology, K.K. Leung Brain Research Centre, The Fourth Military Medical UniversityXi’an, China; ^2^Department of Anatomy, Histology and Embryology, Ningxia Medical UniversityYinchuan, China; ^3^Department of Physiology and Cell Biology, Medical Center, Ohio State UniversityColumbus, OH, USA

**Keywords:** endomorphin-2, myenteric plexus, μ-opioid receptor, inhibitory neuromuscular transmission, colonic motility

## Abstract

The distribution and activity of endomorphins (EMs), which are endogenous μ-opioid receptor (MOR) ligands in the gastrointestinal tract (GI), are yet to be elucidated. The current study aimed to shed light on this topic. EM2 was expressed in the enteric neurons in the myenteric plexus of the mid-colon. Of the EM2-immunoreactive (EM2-IR) neurons, 53 ± 4.6%, 26 ± 4.5%, 26 ± 2.8% and 49 ± 4.2% displayed immunopositive staining for choline acetyl transferase (ChAT), substance P (SP), vasoactive intestinal peptide (VIP) and nitric oxide synthetase (NOS), respectively. A bath application of EM2 (2 μM) enhanced spontaneous contractile amplitude and tension, which were reversed by β-FNA (an antagonist of MOR) but not NG-nitro-L-arginine methyl ether (L-NAME, a non-selective inhibitor of NOS) or VIP_6-28_ (an antagonist of the VIP receptor) in the colonic strips. EM2 significantly suppressed inhibitory junction potentials (IJPs) in 14 of the 17 examined circular muscle cells, and this effect was not antagonized by preincubation in L-NAME. EM2 was widely expressed in interneurons and motor neurons in the myenteric plexus and presynaptically inhibited fast IJPs, thereby enhancing spontaneous contraction and tension in the colonic smooth muscle.

## Introduction

Opioids play a significant role in mediating intestinal smooth muscle activity via the μ-opioid receptor (MOR). Previous studies have shown that MOR is expressed in distinct neuronal populations, including Dogiel type I myenteric neurons such as motor neurons and interneurons responsible for gastrointestinal (GI) motility in the myenteric plexus (Bagnol et al., 1997; Ho et al., 2003; Sternini et al., 2004; DeHaven-Hudkins et al., 2008). The activation of MOR in the enteric nervous system (ENS) leads to decreased GI propulsive activity (Holzer, 2004; Webster et al., 2008). Orally administered morphine, a non-selective exogenous MOR agonist, typically inhibits GI propulsive activity, thereby resulting in constipation (Pappagallo, 2001; Camilleri, 2011; Kapoor, 2012; Labianca et al., 2012). A substantial body of evidence has demonstrated that colonic retention time is 80% of the oroanal transit time in animals, including humans, suggesting that morphine-induced constipation is likely of colonic origin (Banta et al., 1979; Arhan et al., 1981). However, the mechanism underlying this side effect of morphine has yet to be adequately elucidated, and no therapy to antagonize this side effect has been approved to date (Nishiwaki et al., 1998; Holzer, 2007, 2009; DeHaven-Hudkins et al., 2008; Leppert, 2010; Camilleri, 2011; Diego et al., 2011; Wein, 2012; Bader et al., 2013; Rauck, 2013).

Endomorphin-1 (Tyr-Pro-Trp-Phe-Nh2, EM1) and endomorphin-2 (Tyr-Pro-Phe-Phe-Nh2, EM2), the two endogenous ligands of MOR, were isolated from the bovine and human brain in 1997 (Zadina et al., 1997). EM1 and EM2 selectively bind to MOR at a high affinity (Zadina et al., 1997). EM1-immunoreactive (IR) and EM2-IR neuronal cell bodies are principally located in the hypothalamic nuclei, the nucleus tractus solitarii, the dorsal root ganglia (DRG) and the vagal ganglia in the CNS and the peripheral nervous system (PNS; Barr and Zadina, 1999; Martin-Schild et al., 1999; Pierce and Wessendorf, 2000; Niu et al., 2009). Both EM1 and EM2 are involved in the regulation of somatic and visceral information transmission. μ-opioid receptor is the most abundantly expressed opioid receptor in the GI tract, especially the colon (Banta et al., 1979; Nishimura et al., 1986). However, the distributions of EM1 and EM2 in the ENS and their effects on colonic motility are yet to be determined (Sternini et al., 2004). The cellular mechanisms by which MOR modulates intestinal motility also require further elucidation. Our pilot immunofluorescence histochemical staining experiments revealed that myenteric neurons in the rat colon express EM2 but not EM1. Organ bath measurements revealed that exogenous EM2 exerts two direct effects on colonic motility: EM2 enhances spontaneous contractile amplitude and tension and suppresses electrical field stimulation (EFS)-evoked twitch contraction in colonic preparations. Elucidating the mechanism by which EM2 stimulates colonic motility was the primary aim of the present study. We hypothesized that EM attenuates or facilitates colonic motility via the activation of opioid receptors in myenteric nerve cells. Therefore, we investigated the effects of endogenous opioids on colonic motility to enhance our understanding of EM-evoked bidirectional modulatory mechanisms and to provide novel insight into a potential therapeutic strategy in the treatment of GI dysfunction.

## Materials and methods

### Animals

Male Sprague-Dawley rats weighing 220–250 g were provided by the Experimental Animal Center of the Fourth Military Medical University (Xi’an, China). All protocols described below were approved by the Committee of Animal Use for Research and Education of the Fourth Military Medical University. All efforts were made to minimize the number of animals used and their suffering in accordance with the ethical guidelines for animal research (Zimmermann, 1983). After the rats were anesthetized using ether, the mid-colons between the right and left flexures were removed.

### Immunofluorescence histochemistry

For whole-mount colonic preparations, the mucosa, submucosa and inner circular muscle layer from the tissues were separated from the outer longitudinal muscle layer, which was attached to the myenteric plexus.

Whole-mount colonic preparations from seven rats (*n* = 7) were processed for immunofluorescence histochemical double-staining for EM2 and neuron-specific enolase (NSE), choline acetyl transferase (ChAT), substance P (SP), vasoactive intestinal peptide (VIP) or nitric oxide synthetase (NOS, the key enzyme for the synthesis of nitric oxide). The whole-mount sections were sequentially incubated in the following solutions: (1) a mixture of rabbit antiserum against EM2 (AB5104, 1:200; Chemicon, Billerica, MA, USA) and mouse antiserum against NSE (MAB314, 1:200; Chemicon), goat antiserum against ChAT (IHCR1008-6, 1:500; Chemicon), rat antiserum against SP (MAB356, 1:500; Chemicon), mouse antiserum against VIP (SC-25347, 1:200; Santa Cruz, Santa Cruz, CA, USA) or mouse antiserum against NOS (N2280, 1:2000; Sigma, Saint Louis, MO, USA) in 0.01 M phosphate-buffered saline (PBS, pH 7.4) containing 5% (v/v) normal goat serum (NGS), 0.3% (v/v) Triton X-100, 0.05% (w/v) NaN_3_ and 0.25% (w/v) carrageenan (PBS-NGS, pH 7.4) for 72 h at 4°C; (2) a mixture of biotinylated donkey anti-rabbit IgG (BA-1000, 1:200; Vector, Burlingame, CA, USA) for EM2 and Alexa 594-labeled donkey anti-mouse IgG (A21203, 1:500; Invitrogen, Grand Island, NY, USA) for NSE, VIP or NOS, Alexa 594-labeled donkey anti-goat IgG (A-11058, 1:500; Invitrogen) for ChAT- or Cy3-labeled donkey anti-rat IgG (AP189C, 1:200; Chemicon) for SP in PBS-NGS for 6 h at 4°C; and (3) fluorescein isothiocyanate (FITC)-labeled avidin D (A-2001, 1:1,000; Vector) in PBS containing 0.3% Triton X-100 (PBS-X, pH 7.4) for 4 h at room temperature. Finally, the sections were cover-slipped using 0.01 M PBS containing 50% (v/v) glycerin and 2.5% (w/v) triethylene diamine (an anti-fading reagent).

### Control experiments

The rabbit antiserum against EM2 was prepared against the synthetic peptide Tyr-Pro-Phe-Phe-NH2 from full-length EM2 conjugated to BSA. We evaluated the specificity of this antibody by incubating it in either 2 μM EM2 or 2 μM EM1 peptide, as previously described (Huidobro-Toro and Way, 1981; Niu et al., 2009). Normal mouse sera were used to confirm the specificities of the mouse antibodies against NSE, VIP and NOS. Normal goat and rat sera were used to confirm the specificities of the goat antibody against ChAT and the rat antibody against SP, respectively, via replacement tests. In the present study, when the EM2 antibody was pre-absorbed using either homologous or heterologous synthetic peptides and when normal mouse, goat or rat sera were used to replace the mouse, goat or rat antibodies against NSE, VIP and NOS, ChAT and SP, respectively, no immunopositive staining was detected in our preparations. Therefore, the antibodies were considered to be specific and reliable (data not shown).

### Cell counting

After immunofluorescence histochemical staining, all of the fluorescence-labeled sections were observed under a confocal laser scanning microscope (Olympus FV1000; Tokyo, Japan) using the appropriate filters for FITC (excitation 490 nm; emission 520 nm) and Alexa 594 (excitation 590 nm; emission 617 nm). To estimate the extent of co-localization between EM2 and NSE, as well as VIP or NOS, three sets of representative whole-mount sections from the mid-colon were selected (*n* = 7). Plexuses were randomly selected for this analysis from each specimen. A ganglion that displayed a clear boundary and a neuronal cell body outline was randomly selected from each specimen for this analysis. In each specimen, approximately 6–7 ganglia were selected to count the number of various neuronal cell bodies; the total number of ganglia was approximately 35 in each set. Within each plexus, we determined the number of EM2-IR neuronal cell bodies, the number of neuronal cell bodies IR for other markers, and the number and percentage of neuronal cell bodies that expressed both markers.

### Mechanical experiments

The isolated mid-colon (1.0 cm) was rapidly transferred to a dish for dissection. This colon segment was opened along the mesenteric border and pinned (mucosa side up) to a Sylgard base (Dow Corning, Midland, MI, USA). The mucosa layer was removed, and the preparations were sliced into muscle strips in the circular or longitudinal axis. These test strips were placed in 10 ml of organ bath containing modified Krebs solution bubbled with 95% O_2_ and 5% CO_2_ at 37.0 ± 0.5°C, which was replaced every 20–30 min. An isometric force transducer (Harvard VF-1 Harvard Apparatus, Holliston, MA, USA) connected to a computer via an amplifier was used to record the motility of the muscle strips. These data were digitalized (25 Hz) using the Data 2001 software (Panlab, Barcelona, Spain) coupled to an A/D converter and were stored in the computer. The tension was set to 1 g to minimize local reflex stimulation of the bowel, and the strips were allowed to equilibrate for 1 h. Electrical field stimulation (0.5 ms, 10 Hz, and 0.5 mA) was applied using platinum electrodes connected via stimulus isolation units (Grass SIN5) to a square wave stimulator (Grass 588, Grass, Quincy, MA, USA). The concentration of all reagents is presented as the final concentration in 10 ml of organ bath.

### Intracellular microelectrode recordings in circular muscle

The intracellular electrophysiological recording methods were previously described (Wang et al., 2007). In brief, “sharp” glass microelectrodes were used to record stimulus-evoked inhibitory junction potentials (IJPs) in the circular muscle coat of the mucosa-free preparations in which a portion of the circular muscle bands was removed to expose the myenteric plexus for the placement of the stimulation electrode. The preparations were pinned to Sylgard resin at the bottom of a 2.0-ml recording chamber. The chamber was perfused at a rate of 10–15 ml/min with Krebs solution warmed to 37°C and gassed with 95% O_2_–5% CO_2_ to buffer this solution at pH 7.3–7.4. During electrophysiological recording, 1 μM nifedipine and scopolamine were added to the Krebs solution to suppress muscle movements. The microelectrodes, which displayed resistances of 80–120 MΩ, were filled with KCl (2 M) or potassium acetate (4 M). For the intraneuronal injection of electrical current, the preamplifier (M-767; World Precision Instruments, Sarasota, FL, USA) was equipped with a bridge circuit. The Grass SD9 stimulator (Grass Instrument Division, Astro-Med, Warwick, RI, USA) injected rectangular constant-current pulses. The electrometer output was amplified and observed using oscilloscopes (Tektronix 3012, Tektronix, Beaverton, OR, USA). Digital charts of the synaptic events and membrane potentials were recorded on the computer using the Data Acquisition System and LabChart software (ADInstruments, Colorado Springs, CO, USA). The bipolar insulated tungsten stimulating electrodes were connected via stimulus isolation units (Grass SIN5) to the Grass SD9 stimulator and were placed perpendicularly to the longitudinal axis of the preparation. In the myenteric plexus, the focal electrical stimulation of interganglionic fiber tracts induces neuromuscular junction potentials. The stimulus parameters were single pulses at an amplitude of 0.2 mA and a duration of 2 ms.

### Solutions and drugs

The composition of Krebs solution was (in mM): 120 NaCl, 6 KCl, 2.5 CaCl_2_, 1.2 MgCl_2_, 1.35 NaH_2_PO_4_, 14.4 NaHCO_3_, and 11.5 glucose. The reagents used in this study were EM2 and β-FNA hydrochloride (Sigma), NG-nitro-L-arginine methyl ether, hydrochloride (L-NAME, Sigma), nifedipine, scopolamine (Sigma), tetrodotoxin (TTX, Taizhou Kante Biological Co., Ltd., Taizhou, Jiangsu, China), and the VIP fragment VIP_6-28_ (Sigma). The preparation of all stock solutions and their subsequent dilutions was performed using 0.9% saline. All concentrations are expressed as their final concentration in the organ bath and the perfusing chamber for intracellular recording.

### Data analysis

Statistical comparisons of the data sets were performed via ANOVA followed by Dunnett’s *post hoc* test, and “*n*” indicates the number of samples. Student’s *t*-test was used to determine significance, and *P* < 0.05 was considered to be significant.

## Results

### Immunofluorescence histochemical staining

**Figure 1:**
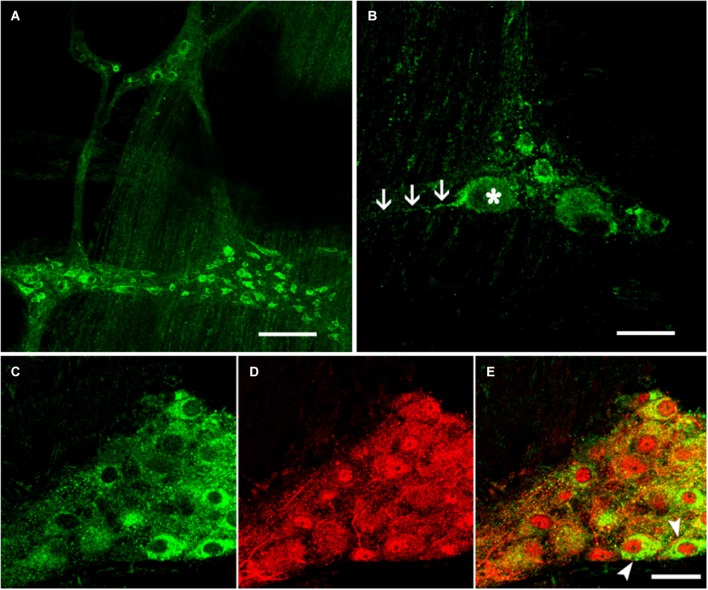
**Immunofluorescence histochemistry of whole-mount sections of the myenteric plexus from the rat colon. (A)** EM2-IR fibers formed a network between the ganglia. **(B)** EM2-IR neurons typically extended a distinctive long process, as indicated by the arrows. Double-label immunofluorescence staining revealed the colocalization of EM2 **(C)** with NSE **(D)**. The composite images show neurons co-labeled with EM2 and NSE **(E)**. The arrows indicate typical neurons that expressed both EM2 and NSE. Scale bar: **A** = 180 μM; **B, C, D** and **E** = 30 μM.

The EM2-IR products were predominantly localized to the cytoplasm of the neuronal cell bodies in the ganglia and in the nerve fibers surrounding the ganglia. EM2 expression was detected at both of these sites in the myenteric plexus of the rat mid-colon. EM2-IR markers were also detected outside the plexus. The EM2-IR nerve fibers formed a dense network between the neuronal cell bodies of the ganglia, and both varicose and non-varicose fibers ran along the internodal strands between the bowel muscle and the myenteric plexus (Figure 1A). The EM2-IR neurons typically displayed an oval cell body in which many thick dendrites protruded from the somata with a long axonal process that extended for a distance of 100 μM or longer. A few EM2-IR neuronal cell bodies outside the primary meshes of the plexus were occasionally identified. The areas of these neuronal cell bodies were 35.5 ± 10.8 μM (length) by 17.8 ± 4.6 μM (width) (Figure 1B). Approximately 10.55 ± 4.2 EM2-IR neurons were detected in each ganglion.

**Figure 2:**
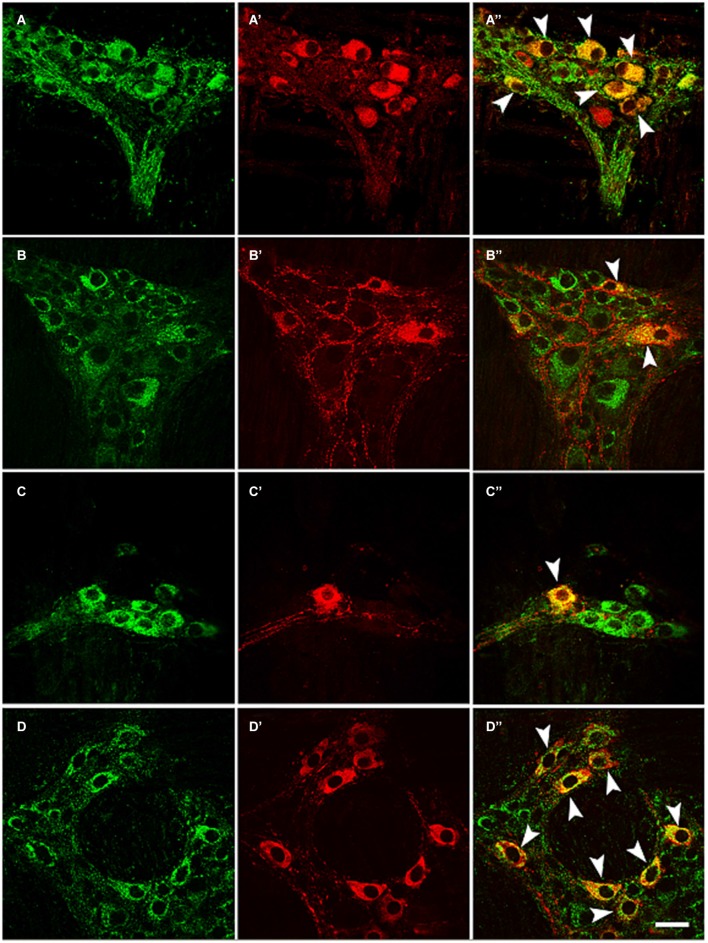
**Double-label immunofluorescence of whole-mount sections showing the colocalization of EM2 (A, B, C, D) with ChAT (A’), SP (B’), VIP (C’) or NOS (D’)**. The composite images show neurons co-labeled with EM2 and ChAT **(A”)**, SP **(B”)**, VIP **(C”)** or NOS **(D”)**. The arrows indicate neurons expressing EM2 and ChAT **(A’)**, SP **(B’)**, VIP **(C’)** or NOS **(D’)**. Scale bar = 30 μM.

Antisera against the general neuronal marker NSE (Mulderry et al., 1988) were used to label all neuronal components of the myenteric plexus, including the neuronal cell bodies and the varicose and non-varicose nerve fibers. Of the NSE-IR neurons, 57% were stained with EM2 in the myenteric ganglia (Figures 1C–E; Table 1). Next, we examined the co-localization between EM2 and ChAT, SP, VIP and NOS. ChAT-IR was predominantly localized to the somata and the nerve fibers, and 53 ± 4.6% of the EM2-IR neuronal cell bodies were co-labeled with ChAT. EM2-IR fibers ran near the ChAT-IR neurons, and some of these fibers were even distributed around the ChAT-IR neuronal cell bodies (Figure 2A; Table 1). SP-IR was intense in the nerve fibers, which ran amid the EM2-IR neuronal cell bodies. Approximately 26 ± 4.5% of the EM2-IR neurons were co-labeled with SP (Figure 2B; Table 1). VIP-IR neuronal cell bodies were occasionally detected in the myenteric plexus. The VIP-IR neurons displayed a slightly oval-shaped cell body and one long process as well as several short and lamellar processes surrounding the perikarya; 26 ± 2.8% of the EM2-IR neuronal cell bodies were co-labeled with VIP (Figure 2C; Table 1). The NOS-IR products displayed a homogeneous distribution in the neuronal cell bodies without nuclear labeling, and 49 ± 4.2% of the EM2-IR neurons were co-labeled with NOS (Figure 2D; Table 1). Based on immunofluorescence histochemical staining (except for that in the EM2-IR neurons), only the NOS-IR processes at their origins could be detected, although these processes may not have extended far. Additionally, some EM2-IR nerve cell bodies were surrounded by VIP-IR and NOS-IR varicosities and terminals (Figures 2A,B). EM2 immunoreactivity was detected in 74 ± 4.7% of the ChAT-IR neurons (Figure 2A; Table 1) and in 88 ± 2.5% of the SP-IR neurons (Figure 2B; Table 1). Among the VIP-IR and NOS-IR neurons, nearly all displayed EM2 immunoreactivity (Figures 2C,D; Table 1).

**Table 1:** **Percentage of the co-localization of EM2 with various markers in myenteric neurons**.

Marker pairs	Numbers of myenteric ganglion neurons (mean ± SD)	Numbers of doubly labeled ganglion neurons (mean ± SD)	Percentages (%) (mean ± S.E.M)
ChAT (n1)/EM2 (n2)	7.23 ± 3.6/9.85 ± 3.8	ChAT of EM2 (n1/n2) EM2 of ChAT (n2/n1)	53 ± 4.6 74 ± 4.7
NOS (n3)/EM2 (n2)	6.67 ± 2.5/13.89 ± 4.8	NOS of EM2 (n3/n2) EM2of NOS (n2/n3)	49 ± 4.2 100.0
NSE (n4)/EM2 (n2)	20.45 ± 5.9/11.45 ± 3.6	NSE of EM2 (n4/n2) EM2 of NSE (n2/n4)	100.0 57 ± 4.1
SP (n5)/EM2 (n2)	2.62 ± 1.3/8.77 ± 2.9	SP of EM2 (n5/n2) EM2 of SP (n2/n5)	26 ± 4.5 88 ± 2.5
VIP (n6)/EM2 (n2)	2.5 ± 0.8/10.4 ± 3.8	VIP of EM2 (n6/n2) EM2 of VIP (n2/n6)	26 ± 2.8 100.0

### Effects of EM2 on colonic motility

**Figure 3:**
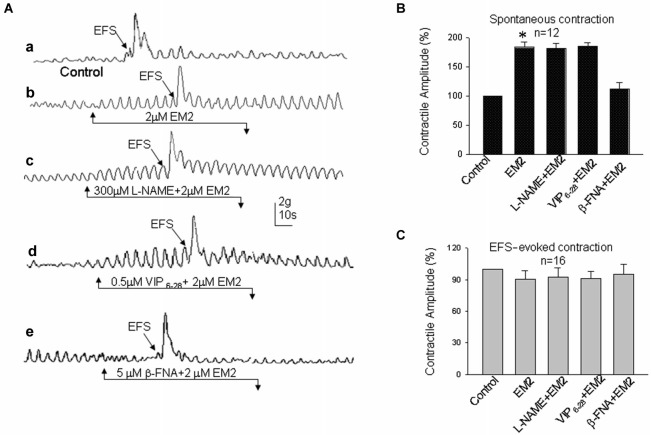
**Effects of EM2 on the motility of circular muscle strips from the colon. (A)** Spontaneous contractile amplitude and EFS-evoked contractions **(a)**. A bath application of EM2 enhanced the spontaneous contractile amplitude and slightly suppressed EFS-evoked contractions **(b)**. The presence of both L-NAME (an antagonist of NO) and VIP_6-28_ (a VIP receptor antagonist) did not significantly alter the effects of EM2 **(c,d)**. β-FNA (an antagonist of MOR) blocked the EM2-induced changes in the strips **(e). (B)** Summary of the effects of EM2 on spontaneous contractions. **(C)** Summary of EFS-evoked contractions. *P < 0.05.

**Figure 4:**
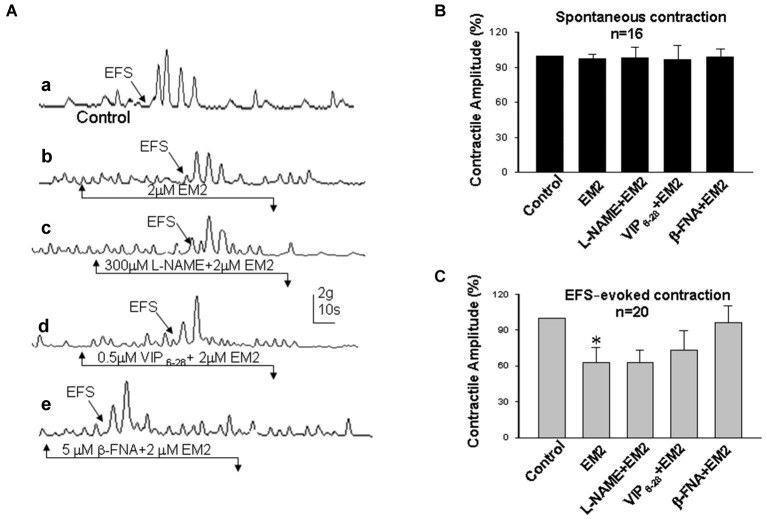
**Effects of EM2 on the motility of longitudinal muscle strips from the colon. (A)** Spontaneous contractile amplitude and electrical field stimulation (EFS)-evoked contractions **(a)**. Exposure of the strips to EM2 inhibited EFS-evoked contraction but did not appear to affect the spontaneous contractile amplitude **(b)**. The presence of both L-NAME and VIP_6-28_ did not significantly alter the effects of EM2 **(c,d)**. β-FNA blocked the EM2-induced changes in the strips **(e). (B)** Summary of the effects of EM2 on spontaneous contractions. **(C)** Summary of EFS-evoked contractions. *P < 0.05.

The colonic muscle strips were mounted in an organ bath chamber to determine the effects of EM2 on intestinal motility. Regular cyclic spontaneous contractions occurred after incubation in Krebs solution for one half hour (24 preparations/6 rats). Bath application of EM2 (2 μM) enhanced the amplitude of the spontaneous contractile waves in six circular and six longitudinal muscle strips (Figures 3, 4A,B). The positive effects of EM2 on spontaneous contractions were not altered in the presence of 300 μM L-NAME, a non-selective inhibitor of NO synthase, or 0.5 μM VIP_6-28_, a VIP receptor antagonist. The application of the selective MOR antagonist β-FNA (5 μM) reversed the positive effects of EM2 on colonic contraction (Figures 3, 4A,B). Tetrodotoxin (1 μM), a Na^+^ channel blocker, completely blocked the effects of EM2 on the muscle strips but partially suppressed spontaneous motility (results not shown).

Electrical field stimulation evoked twitch contractile waves in the colonic strips (Figures 3, 4Aa). The exposure of the strips to EM2 (2 μM) partially suppressed the EFS-evoked contractile waves (*n* = 16; Figures 3, 4Ab). The depressive effect of EM2 on the EFS-evoked contractile waves was abolished by β-FNA (5 μM; Figures 3, 4Ae,C). Preincubation in either L-NAME (300 μM) or VIP_6-28_ (0.5 μM) did not significantly alter the suppressive effects of EM2 on EFS-evoked twitch contraction (Figures 3, 4Ac,d,C).

### Intracellular recordings in circular muscle cells

**Figure 5:**
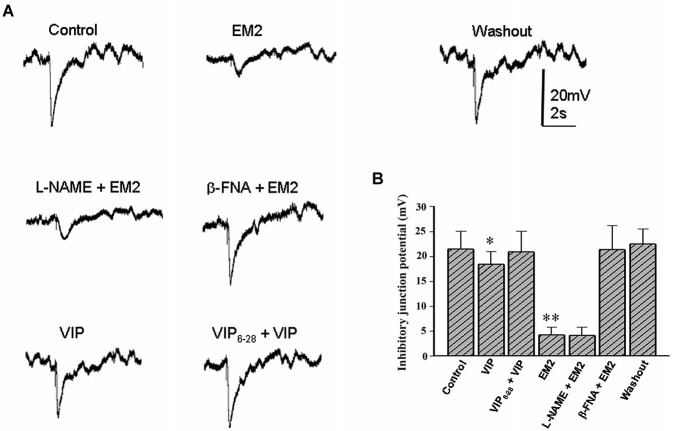
**EM2 inhibits inhibitory neuromuscular transmission in the circular muscle of the colon. (A)** A bath application of EM2 significantly suppressed IJPs. The effect of EM2 on the IJPs was antagonized *via* pretreatment with β-FNA but not L-NAME. The application of VIP slightly reduced the IJPs, which was reversed *via* pretreatment with VIP_6-28_. **(B)** Summary of the inhibitory effect of EM2 on electrical stimulation-evoked IJPs in the circular muscle of the colon. *P < 0.05 and **P < 0.01.

To explore the cellular mechanisms by which EM2 modulates colonic motility, intracellular recordings were performed to examine the synaptic responses of neuromuscular junctions and the potential changes in the circular muscle of seven colonic myenteric preparations. Both spontaneous excitatory junction potentials (EJPs) and IJPs were detected in the colonic circular muscle cells in the presence of Krebs solution lacking nifedipine (an L-type calcium channel blocker) and scopolamine (a competitive antagonist of M-type muscarinic receptors). Perfusion with EM2 (2 μM) suppressed either EJPs or IJPs in 22/26 circular muscle cells from nine colonic myenteric preparations. Nevertheless, the recordings did not remain stable in the muscle cells for an extended period (commonly <10 min) because strong muscle movement resulted in the dislocation of the microelectrode from the recorded muscle cells. Under these recording conditions, EFS also evoked rapid and strong movements that terminated the recordings. Because previous evidence has demonstrated that opioids inhibit intestinal movement *via* presynaptic MORs to reduce acetylcholine release (Fichna et al., 2007), the present investigation focused on the effects of EM2 on inhibitory neuromuscular transmission in these muscle cells. When Krebs solution containing nifedipine (1 μM) and scopolamine (1 μM) was employed to inhibit muscle movement, EFS-evoked IJPs were stably recorded from the circular muscle cells. The application of EM2 (2 μM) significantly suppressed the amplitude of the EFS-evoked IJPs at an IC_50_ of 0.35 ± 0.13 μM (*n* = 16; Figures 5A,B). The pre-application of β-FNA (10 μM) markedly ameliorated the inhibitory effect of EM2 on these IJPs (Figures 5A,B). However, the application of β-FNA (10 μM) alone did not exert any clear effect on the IJPs. To examine the effects of EM2 on the nitric oxide- and VIP-mediated slow phase of the IJPs, the NOS inhibitor L-NAME and the VIP receptor antagonist VIP_6-28_, respectively, were used. Neither L-NAME (300 μM) nor VIP_6-28_ (0.2 μM) exerted a significant effect on the EM2-mediated inhibition of the IJPs (Figures 5A,B).

## Discussion

Neurons in the myenteric plexus are located between the circular and longitudinal muscle layers. Myenteric neurons extend projection fibers to the circular and longitudinal muscle layers (Grider and Foxx-Orenstein, 1999), forming a widely distributed neuronal network that regulates intestinal muscle tension and propulsion (Sarna, 2006). Gastrointestinal tract motility disorders induced by opioids are likely of colonic origin and mediated *via* MOR, such that EM, a potent endogenous MOR agonist, may participate in the modulation of GI motility. However, the mechanisms by which EM modulates GI motility are yet to be identified despite recent morphological and functional findings (Fichna et al., 2007). In the present study, we tested the hypothesis that EM inhibits colonic motility *via* the activation of opioid receptors in myenteric neurons by combining immunostaining, organ bath and intracellular recording methods to obtain morphological, physiological and pharmacological data. It is helpful to understand the mechanism by which EM2 modulates colonic motility. The major findings in the present study include the expression of EM2 in myenteric neurons and the EM2-mediated suppression of colonic motility *via* the activation of presynaptic MORs to inhibit inhibitory and excitatory neuromuscular transmission.

### Distribution and characteristics of EM2-IR neurons in the myenteric plexus of the rat colon

In the present study, immunofluorescence histochemical staining revealed that most (57%) EM2-IR neuronal cell bodies and fibers formed a dense network that was restricted to the colonic myenteric plexus. These EM2-IR neurons are also immunopositive for ChAT, SP, VIP or NOS.

Previous studies showed that ChAT-expressing neurons correspond to ascending excitatory motor neurons that innervate the muscle and that some populations of ascending and descending interneurons also express ChAT (Costa et al., 1996). Substance P acts on smooth muscle directly or indirectly by modulating the release of excitatory transmitters from nerve fibers (Scheurer et al., 1994). VIP-IR is localized to both ascending and descending interneurons, as well as to descending inhibitory motor neurons (Costa et al., 2000; Lomax and Furness, 2000; Smith et al., 2007). Additionally, NOS has been detected in both circular and longitudinal inhibitory motor neurons and in descending interneurons (Schemann and Neunlist, 2004). ChAT-IR, VIP-IR and NOS-IR neurons in the myenteric plexus (Fickel et al., 1997; Benabdallah et al., 2008) but not the smooth muscle of the rat intestine (Fickel et al., 1997; Porcher et al., 1999; Fichna et al., 2007) expressed MORs (Fickel et al., 1997; Ho et al., 2003). At the cellular level, MORs are predominantly localized to the cell surface of motor neurons and interneurons and to the nerve fibers that innervate the longitudinal and circular muscles (Ho et al., 2003). Our morphological findings indicated that EM2 colocalized with both VIP and NO in the perikarya of myenteric neurons, suggesting that EM2-IR neurons correspond to a subpopulation of myenteric interneurons and/or motor neurons.

### Effects of EM2 on colonic motility

In the current study, we demonstrated that EM2 partially inhibited EFS-evoked contraction waves and that this was reversed by the MOR antagonist β-FNA. Enteric excitatory motor neurons are commonly thought to release excitatory transmitters, such as cholinergic and tachykininergic neurotransmitters (Nedialkova et al., 2011). The finding that EM2 suppresses EFS-evoked colonic twitch contraction also suggests that EM2 plays an inhibitory role in the release of excitatory transmitters from these motor neurons.

However, EM2 enhanced the spontaneous contractile amplitude, which was also reversed by the MOR antagonist β-FNA. This finding suggests that EM2 directly activates MORs on motor neurons to suppress neurotransmitter release into the colonic strips. We also found that TTX, a neuronal Na^+^ channel blocker, completely blocked the effects of EM2 on the muscle strips but did not abolish its effects on spontaneous contraction, indicating that the effects of EM2 on colonic motility involve neurogenic modulation. The spontaneous contractions of colonic segments are induced and maintained by an increase in the tonus (Benabdallah et al., 2008), which is regulated by non-cholinergic non-adrenergic neurotransmitters, including NO and VIP (Benabdallah et al., 2008). Tonically released NO from myenteric neurons inhibits increases in spontaneous motility both *in vitro* and *in vivo* (Gil et al., 2010), and VIP inhibits the spontaneous mechanical activity induced by the cyclic depolarization of the circular muscle (Plujà et al., 2000). The NO-induced inhibitory tone contributes to rhythmic contractions, which produce the mixing and propulsive movements in the fasting and the postprandial states (Sarna, 2006). L-NAME and VIP_6-28_ exerted no clear effect on the EM2-induced enhancement of spontaneous muscle strip contractions. These results suggest that the effect of EM2 on colonic motility is not due to the neuronal or muscular activities of NO or VIP. Accumulating evidence has demonstrated that non-cholinergic non-adrenergic inhibitory neuromuscular transmission contains purinergic, nitrergic and VIP-ergic components and that the purinergic component plays the primary role in inhibiting transmission (Sarna, 2006; Wang et al., 2007).

μ-opioid receptors are known to modulate pre-and post-synaptic neurotransmission, thereby inhibiting neural activity in the ENS. The present study is the first to report that EM2 activates MORs to enhance colonic motility in rats; this is consistent with other findings from mice (Yu et al., 2007; Wang et al., 2008). The dual effects of EM2 to attenuate and facilitate bowel contractions suggest that EM2 performs a bidirectional modulation of colonic motility.

### Intracellular microelectrode recordings in circular muscle

The electrophysiological results of the present study demonstrated that exogenous EM2 significantly suppressed fast IJPs but did not significantly affect slow IJPs. The pre-application of the selective MOR antagonist β-FNA reversed the inhibitory effects of EM2 on the IJPs. Immunohistochemical evidence indicates that MORs are expressed in myenteric neurons (Fickel et al., 1997; Ho et al., 2003) but not in intestinal smooth muscle (Fickel et al., 1997; Porcher et al., 1999). Previous morphological evidence is consistent with our results that EM2 exerts no direct effect on smooth muscle in the bowel but plays a neurogenic regulatory role in intestinal motility. The activation of presynaptic MORs has been reported to inhibit the release of acetylcholine and ATP in the intestinal submucosal plexus (Yun et al., 2004). We found that EM2 not only inhibits EFS-evoked fast and strong contractions but also enhances spontaneous contractile waves and tension in the bowel strips. Elucidating this facilitative effect of EM2 and its underlying mechanism were the primary aims of the present study. To explore these cellular mechanisms, neuropharmacology and electrophysiological recording were performed to examine neuromuscular transmission in the rat mid-colon. Pre-incubation in the MOR antagonist β-FNA specifically reversed this inhibitory effect of EM2. The present electrophysiological and neuropharmacological results demonstrated that the facilitative effect of EM2 on colonic motility occurs *via* a presynaptic mechanism, i.e., the suppression of the release of inhibitory transmitters, to augment smooth muscle contraction and tension. Previous evidence has shown that IJPs are generally biphasic and consist of an initial large-amplitude and rapidly activating component (fast IJP) followed by a smaller and more slowly activating component (slow IJP). The slow IJP is abolished by treatments that block the synthesis of nitric oxide. However, purinergic neurotransmitters (*via* activating P2Y_1_ receptors) are responsible for the primary fast component of non-cholinergic non-adrenergic IJPs (Wang et al., 2007; Gallego et al., 2012; Hwang et al., 2012; Goyal et al., 2013).

Taken together, the present results indicate the following: (1) EM2 acts as an endogenous opioidergic neurotransmitter in the rat colon; (2) EM2 activates MORs that are localized to ENS neurons and nerve terminals in the smooth muscle coat to modulate colonic motility; and (3) EM2 inhibits EFS-evoked muscle contractions, potentially by suppressing the release of prejunction excitatory neurotransmitters, and suppresses inhibitory ligand release to enhance spontaneous colonic contractions and tension. These inhibitory ligands do not include NO or VIP but may include purinergic inhibitory transmitters.

In conclusion, EM2-IR was found to be widely localized to the cell bodies of enteric neurons that also display immunoreactivity for ChAT-, NOS-, SP- or VIP in the myenteric plexus of the rat colon. EM2, an endogenous opioid, inhibits intestinal motility in accordance with our hypothesis and enhances colonic smooth muscle movement by decreasing or reducing inhibitory transmitter release into the bowel neuromuscular junction. This EM2-mediated bidirectional modulation of colonic motility may provide new insight into the mechanisms underlying GI protection and dysfunction, which may help us to establish a novel understanding of the opioidergic system in the GI tract and new therapeutic strategies for GI diseases.

## Authors’ contributions

Morphological experiments: Jun-Ping Li, Chang-Jun Gao, Yong-Hui Liao, Juan Qu and Zhong-Yi He. Electrophysiological experiments: Jun-Ping Li, Xi-Yu Wang and Guo-Du Wang. Technical support: Ting Zhang. Manuscript writing: Jun-Ping Li, Guo-Du Wang and Yun-Qing Li. Study conception and design: Yun-Qing Li.

## Conflict of interest statement

The authors declare that the research was conducted in the absence of any commercial or financial relationships that could be construed as a potential conflict of interest.
